# First verified record of the ant genus *Calyptomyrmex* (Hymenoptera: Formicidae) from India, along with a revised key to known Indomalayan species

**DOI:** 10.3897/BDJ.3.e5420

**Published:** 2015-11-16

**Authors:** Shahid Ali Akbar, Himender Bharti

**Affiliations:** ‡ Punjabi University, Patiala, India

**Keywords:** Cryptic, Formicidae, *
Calyptomyrmex
*, Kerala, India

## Abstract

**Background:**

The members of genus *Calyptomyrmex* are mostly encountered under rotten logs, in the soil, under stones and in leaf litter samples. These ants are seldom in collections making estimation of their true distributional patterns problematic (Shattuck 2011). The deep antennal scrobes and the unique configuration of the clypeus are distinct to the genus (Bolton 1981).

**New information:**

Herein *Calyptomyrmex wittmeri* Baroni Urbani, 1975 is redescribed and reported for the first time from India. This also confirms the first valid published record of the genus from the country. The image hosted by AntWeb as *C. vedda* (CASENT0280817; AntWeb 2015b) collected by Besuchet, Löbl, Mussard from Kerala, India and identified by Brown is actually *C. wittmeri* (Brown was uncertain of his determination of *C. vedda* and cautiously inserted an interrogation point in front of his determination). Two workers recently collected at Salim Ali Bird Sanctuary, Kerala present similarities to the specimen identified by Brown. However, characters as the lack of well-developed promesonotal suture, absence of clavate setae, and narrow petiolar node, concur with the diagnosis of *C. wittmeri*. A revised key to known Indomalayan species of the genus is provided herewith.

## Introduction

*Calyptomyrmex* is distributed throughout the Afrotropical, Malagasy, Indomalaya, and Australasia regions (Bolton 2015, AntWeb 2015a, Baroni Urbani 1975, Bolton 1981, Shattuck 2011). Most of the species of the genus have restricted distributional ranges, and are found rarely in samples (Bolton 1981​). At present, 37 valid species are recognized (Bolton 2015). These ants have bizarre body pilosity which seems to help in camouflage by retention of soil particles within. This convergent behavior is also shared among other distinct species groups (Bolton 1981, Hölldobler and Wilson 1986). The genus awaits a global taxonomic revision; significant contributions to the genus include Shattuck (2011) revision of Australasian species of the genus, adding 14 new species. Prior to this, Bolton (1981) revised the *Calyptomyrmex* fauna of the Afrotropical region describing seven new species and Baroni Urbani (1975) revised the genus from the Indomalayan region describing four new species, and reported the genus for the first time from Indian subcontinent.

## Materials and methods

The specimens were collected using Winkler extraction method. Sampling was carried out in Salim Ali Bird Sanctuary, which constitutes an important part of Western Ghats of India. The region is known to harbor several rare and endemic ant species (Bharti and Akbar 2013a, Bharti and Akbar 2013b, Bharti and Akbar 2013c, Bharti and Akbar 2014a, Bharti and Akbar 2014b, Bharti and Akbar 2014c, Bharti and Akbar 2014d). The taxonomic analyses were carried out using Nikon SMZ 1500 stereo zoom microscope. For digital images, an MP evolution digital camera was used on same microscope with Auto-montage (Syncroscopy, Division of Synoptics, Ltd.) software. Later, images were cleaned as per requirements with Adobe photoshop CS6. Description patterns and morphological terminology are detailed below. Measurements (given in millimeters) follow Baroni Urbani (1975), Bolton (1981), Shattuck (2011) include: **CFW** Clypeal fork width measured as maximum width between fork-like cuticular projection on the anterior-most part of the clypeus; **HL** Head length is measured from the base of the clypeal fork to the mid-point of the occipital margin, in a straight line in full-face; **HW** Maximum head width in full face view; **CI** (Cephalic index) = HW/HL x 100; **SL** Length of the scape (basal-most antennal segment) excluding the basal constriction and condylar bulb; **SI** Scape index = SL/ HW x 100; **PetL** Maximum length of the petiolar node in dorsal view; **PetW** Maximum width of the petiolar node in dorsal view; **PetI** Petiolar index = PetW/PetL x 100; **PronW** Maximum width of the pronotum in dorsal view.

Glossary of terminology:

**Clypeal lamella:** narrow and thin transverse strip of cuticle found along the anterior clypeal border; also referred to as the clypeal apron or clypeal bilobed fork.

**Clypeus:** in dorsal view, the anterior sclerite of the head, which consists of narrow lateral portions and a shield-like median portion.

**Full-face view:** orientation of the head in which the midpoint of the anterior clypeal margin, the mid point of the posterior margin, and the midpoints of the sides are in focus at the same time.

**Gaster:** abdominal segments 3-7 when only petiole is present or abdominal segments 4-7 when both petiole and postpetiole are present; functionally, the terminal, enlarged tagma of the body.

**Mesosoma:** the second main body division of an ant. Morphologically, it is composed of the three thoracic segments (pro-, meso-, and metathorax) to which is fused the propodeum.

**Metanotal groove:** a transverse suture between the mesonotum and the propodeum.

**Ommatidium:** an individual facet of the compound eye.

**Petiole: in Myrmicinae,** the anterior-most of the two segments separating the mesosoma and the gaster. This is one of the defining features of ants. Morphologically, it is the second segment of the abdomen.

**Postpetiole:** morphologically, the third abdominal segment. In strict usage the term postpetiole should only be applied when the third abdominal segment is reduced and separated from the petiole anteriorly and the fourth abdominal segment posteriorly.

**Promesonotal suture:** the transverse suture across the dorsal face of the mesosoma that separates the pronotum from the mesonotum.

**Propodeum:** the dorsal posterior plate of the mesosoma. Morphologically, it is the first segment of the abdomen, fused to the thorax. It may have specializations such as spines, teeth, or lobes.

**Punctate:** describes surface sculpturing composed of round pits, which may be shallow or deep.

**Striate:** describes sculpturing composed of shallow, parallel grooves or lines.

## Taxon treatments

### Calyptomyrmex wittmeri

Baroni Urbani, 1975

Calyptomyrmex wittmeri Baroni Urbani, 1975 - Baroni Urbani (1975): 396, figs. 1, 5 (w.) BHUTAN​.

#### Materials

- **Type status:** Holotype. **Occurrence:** recordedBy: ​C. Baroni Urbani, O. Stemmler, W. Wittmer, M. Würmli; individualCount: 1; sex: worker; lifeStage: adult; **Taxon:** scientificName: Calyptomyrmex wittmeri; **Location:** country: Bhutan; locality: Phuntsholing, Thimphu km 14; georeferenceProtocol: label; **Identification:** identifiedBy: C. Baroni Urbani; dateIdentified: 1975; **Event:** eventDate: Feb-12-1970; **Record Level:** language: Italian; collectionCode: Insects; ownerInstitutionCode: MHMBXXI.V.d.36333, Basel, Switzerland; basisOfRecord: PreservedSpecimen
- **Type status:** Paratype. **Occurrence:** catalogNumber: CASENT0900994; recordedBy: C. Baroni Urbani, O. Stemmler, W. Wittmer, M. Würmli; individualCount: 1; sex: worker; lifeStage: adult; **Taxon:** scientificName: Calyptomyrmex wittmeri; **Location:** country: Bhutan; locality: Phuntsholing, Thimphu km 14; georeferenceProtocol: label; **Identification:** identifiedBy: C. Baroni Urbani; dateIdentified: 1975; **Event:** eventDate: Feb-12-1970; **Record Level:** language: Italian; collectionID: ANTC20153; collectionCode: Insects; ownerInstitutionCode: BMNH#1014394, London, U.K.; basisOfRecord: PreservedSpecimen; source: https://www.antweb.org/specimen/CASENT0900994
- **Type status:** Other material. **Occurrence:** catalogNumber: CASENT0280817; recordedBy: Besuchet, Löbl, Mussard; individualCount: 1; sex: worker; lifeStage: adult; **Location:** country: India; stateProvince: Kerala; locality: Cardamon Hills, Valara Falls, 46 km SW Munnar; verbatimElevation: 450m; georeferenceProtocol: label; **Identification:** identifiedBy: W. L. Brown; dateIdentified: 1972; **Event:** eventDate: 25/11/1972; **Record Level:** language: en; collectionID: ANTC19915; collectionCode: Insects; ownerInstitutionCode: BMNH#1017692, London, U. K.; basisOfRecord: PreservedSpecimen; source: https://www.antweb.org/specimen/CASENT0280817
- **Type status:** Other material. **Occurrence:** recordedBy: Shahid A. Akbar; individualCount: 3; sex: worker; lifeStage: adult; **Taxon:** scientificName: Calyptomyrmex wittmeri; **Location:** country: India; stateProvince: Kerala; locality: Salim Ali Bird Sanctuary; verbatimElevation: 50m; **Identification:** identifiedBy: H. Bharti & Akbar, S.A.; dateIdentified: 2011; **Event:** samplingProtocol: Winkler extraction; eventDate: 06/11/2011; **Record Level:** language: en; collectionCode: Insects; ownerInstitutionCode: PUAC; basisOfRecord: PreservedSpecimen

#### Description

Worker (Fig. 1).

**Worker measurements:** CFW 0.11–0.12; CI 92.75–93.05: HL 0.61–0.64; HW 0.67–0.68; PetI 134–136; PetL 0.16–0.20; PetW 0.24–0.27; PronW 0.31–0.39; SI 50–52; SL 0.24–0.26.

Head globular in full face view, with posterior margin more or less rounded. Clypeus with a broad bilobed fork-like cuticular projection on its anterior-most part. Masticatory margin with 5-teeth. Eyes with 6-8 ommatidia in greatest diameter. Antennae 12-segmented with 3-segmented club.

Mesosoma short and stubby, forming a continuous arch in profile;promesonotal suture not distinct in dorsal view. Metanotal groove indistinct. Propodeal declivity concave. Propodeum in lateral view with broad triangular spines, somewhat divergent in dorsal view. Petiole penduculate, with stocky petiolar node. In dorsal view, anterior face of petiolar node straight while posterior face slightly concave in the middle.

Gaster rounded; truncated anteriorly.

Mandibles' dorsal face striate along entire length. Dorsum of head having confused network of striations. Underlying surface distinctly punctuate. Mesosoma, petiolar and postpetiolar nodes with irregular, widely spaced striations, which are more prominent along sides of pronotum and dorsal surface of petiole. Gaster with a fine matte appearance.

Hairs on head and body erect, slightly broader in posterior half with sharp or blunt tips.

Colour dark-brown, the antennae and legs slightly lighter.

#### Diagnosis

The species is distinct in having body covered with hair only sharp or truncated, but virtually never dilated in any way (Baroni Urbani 1975).

#### Distribution

India (Southern India), Sri Lanka.

#### Ecology

Specimens of this species were collected through Winkler extraction method from Salim Ali Bird Sanctuary, Kerala.

#### Biology

The specimens were encountered in leaf-litter samples. Little is known about the biology of the ant.

## Identification Keys

### Key to Indomalayan species of *Calyptomyrmex*

**Table:** 

1	Dorsal face of the head with clavate, spatular, or spoon-shapped setae	2
–	Dorsal face of the head with erect, much thinner setae	8
2	Presence of propodeal spines or short spiniform projections	3
–	Absence of propodeal spines or short spiniform projections	7
3	Propodeal spines well-developed	4
–	Propodeal spines as short spiniform projections	6
4	First gastral tergite with spoon-shaped/clavate setae. In dorsal view, promesonotal suture present or absent. Variable number of mandibular teeth	5
–	Thinner spatulate setae on the first astral tergite. In dorsal view, promesonotal suture visible. Six mandibular teeth. Color light-brown (Sri Lanka)	*C. vedda*
5	Promesonotal suture visible in dorsal view. First gastral segment finely punctate. Dorsal face of petiole and postpetiole punctate. Color brown (Bhutan)	*C. friederikae*
–	Promesonotal suture not visible in dorsal view. First gastral tergite with deeply impressed puctations. Dorsal face of petiole and postpetiole reticulate. Color brown (Sri Lanka)	*C. tamil*
6	Petiolar node, in profile, subquadrate. Abundant spoon-shaped/clavate setae on the dorsal face of the body. Row of thick spoon-shaped/clavate setae on the lateral margins of the propodeal dorsal face. Setae absent on the anterior half of the first gastral sternite. In dorsal view, petiolar node narrower than postpetiole. Color red-brown (Borneo, Indonesia, Malaysia)	*C. danum*
–	Petiolar node, in profile, round. Sparser, thinner setae on the dorsal face of the body. Spatular setae absent on the majority of propodeal dorsal face. Thin, spatular setae present on the anterior half of first gastral sternite. In dorsal view, petiolar node wider than postpetiole. Color light-brown (Sri Lanka)	*C. singalensis*
7	Posterior margin of head above the level of antennal scrobe narrower, and the lateral corners more rounded(HW < 1.10mm); body smaller (mesosoma length: 0.91–1.02, provided by Shattuck 2011); spatulate hairs narrower and more linear. Color red-brown to light red-brown (New Guinea, Borneo, Indonesia, Malaysia, Philippines, Singapore, Thailand, Australia)	*C. beccarii*
–	Posterior margin of head above the level of antennal scrobes broader, the lateral corners more angular (HW >1.13mm); body larger (mesosoma length: 1.05–1.14 provided by Shattuck 2011); spatulate hairs broader and more rounded. Color red-brown (Borneo, Indonesia, Malaysia, Philippines)	*C. loweryi*
8	In lateral view, dorsal margin of the mesosoma continuous	9
–	In lateral view, dorsal margin of the mesosoma discontinuous	11
9	Postpetiole with spine-like antero-ventral projection. Compound eyes small (2–3 ommatidia in greatest diameter). Propodeum, in lateral view, armed with moderately long, narrow spines. Color light-brown (Vietnam)	*C. rectopilosus*
–	Postpetiole without antero-ventral projection. Compound eyes larger (more than 5 ommatidia in greatest diameter). Propodeum, in lateral view, armed or not with spines. Color variable	10
10	Propodeal spines well-developed. First gastral segment finely reticulate. Petiolar peduncle comparatively long and thin. Petiolar node round in lateral view. Color light-brown (Bhutan, China, India)	*C. wittmeri*
–	Propodeal spines as short, round projections. First gastral segment heavily striate longitudinally. Petiolar peduncle shorter and thicker. Petiolar node subquadrate in lateral view. Color red-brown (Malaysia, Borneo, Indonesia)	*C. retrostriatus*
11	Propodeal spines short, but well-developed. Setae on leading edge of scape long, about as long as scape width. Comparatively long setae on the dorsal face of head, mesosoma, petiole, and gaster. In lateral view, petiolar node with angulate dorso-posterior corner. Color red-brown (Borneo, Indonesia, Malaysia)	*C. sabahensis*
–	Propodeal spines as short, round projections. Setae on leading edge of scape long, about as long as scape width. Shorter setae on the dorsal face of head, mesosoma, petiole, and gaster. In lateral view, petiolar node with angulate dorso-posterior corner. Color red-brown	12
12	Lateral face of mesosoma and dorsal face of head with few, well-defined, broad rugae. In lateral view, petiolar node with strongly carinate dorso-posterior corner. In lateral view, the highest part of the mesosoma is the posterior half of the pronotal dorsal margin (Borneo, Indonesia, Malaysia)	*C. asper*
–	Lateral face of mesosoma and dorsal face of head with numerous, ill-defined, narrower rugae. In lateral view, petiolar node with angulate dorso-posterior corner, but never strongly carinate. In lateral view, the highest part of the mesosoma is the entire pronotal dorsal margin (the entire dorsal margin of the pronotum has the same height) (Borneo, Indonesia, Malaysia)	*C. ryderae*

## Supplementary Material

XML Treatment for Calyptomyrmex wittmeri

## Figures and Tables

**Figure 1a.**
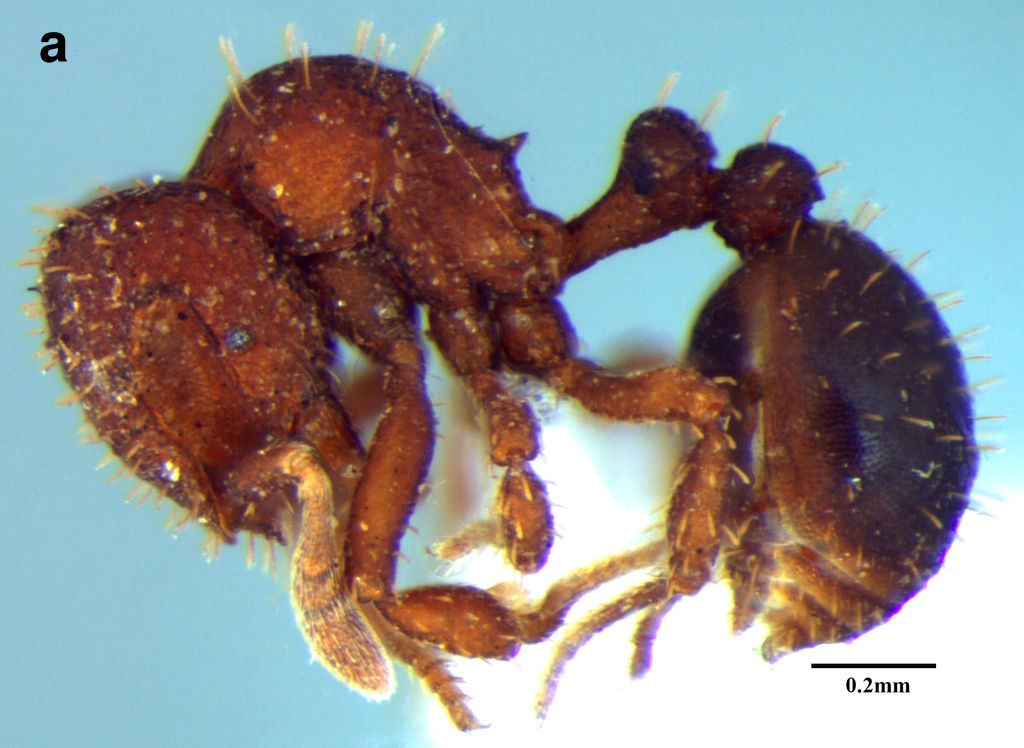
Body in lateral view

**Figure 1b.**
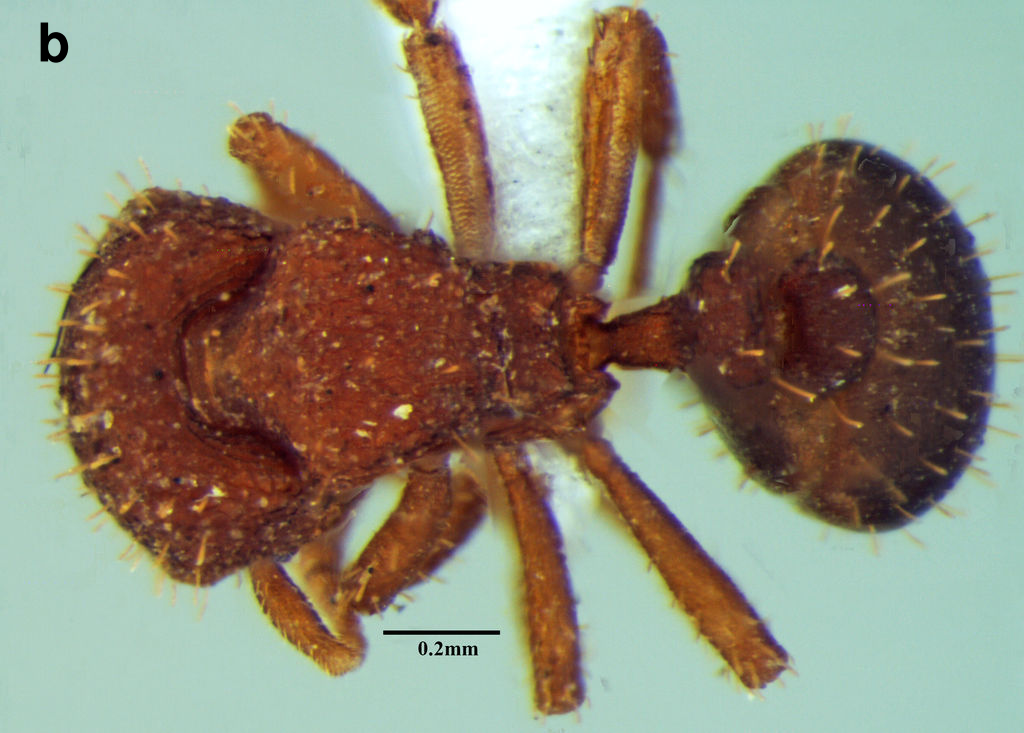
Body in dorsal view

**Figure 1c.**
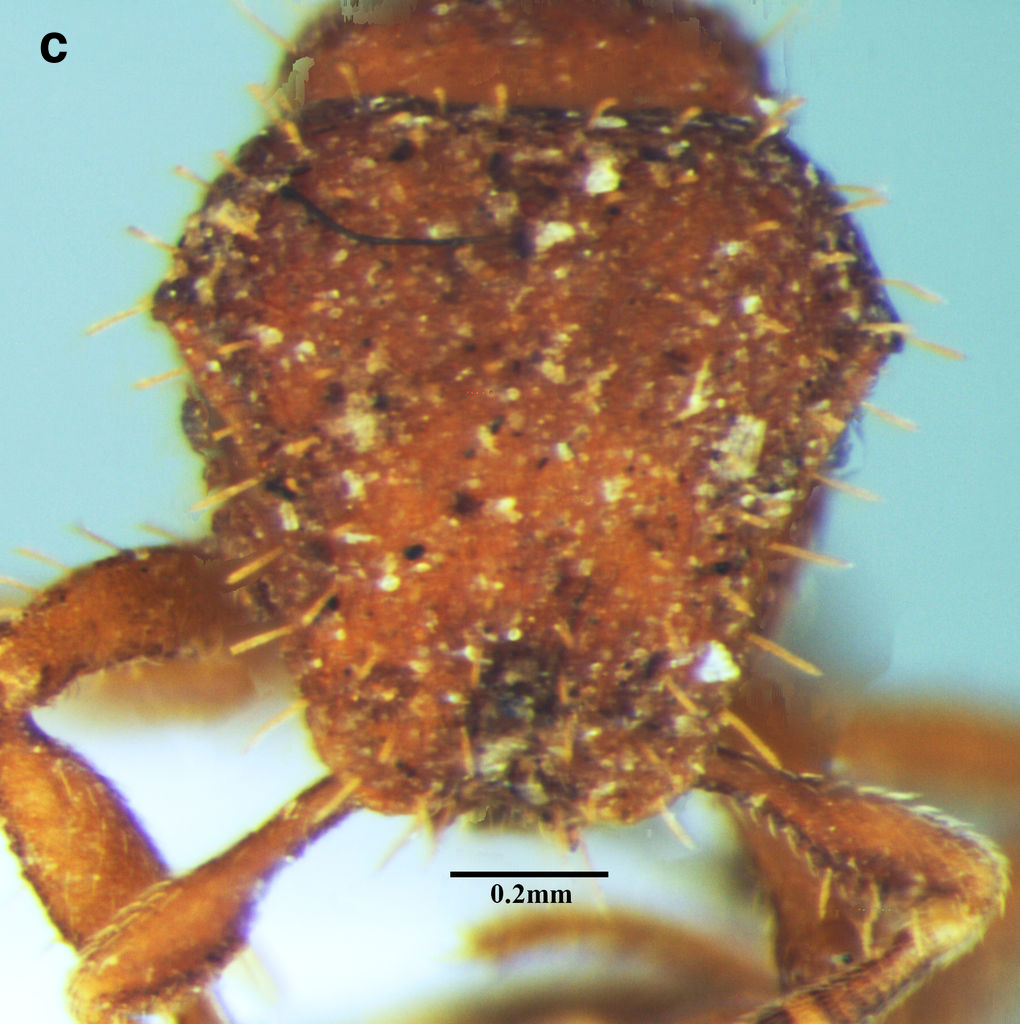
Head in full-face view
